# Barriers and Facilitators in the Implementation of Prevention Strategies for Chronic Disease Patients—Best Practice GuideLines and Policies’ Systematic Review

**DOI:** 10.3390/jpm13020288

**Published:** 2023-02-04

**Authors:** Marta Duda-Sikuła, Donata Kurpas

**Affiliations:** 1 Clinical Trial Department, Wroclaw Medical University, 50-556 Wroclaw, Poland; 2 Department of Family Medicine, Wroclaw Medical University, 51-141 Wroclaw, Poland

**Keywords:** chronic disease, prevention and control, practice guidelines, policy, personalized medicine

## Abstract

Visits of chronically ill patients account for 80% of primary care consultations. Approximately 15–38% of patients have three or more chronic diseases, and 30% of hospitalisations result from the deteriorating clinical condition of these patients. The burden of chronic disease and multimorbidity is increasing in combination with the growing population of elderly people. However, many interventions found to be effective in health service studies fail to translate into meaningful patient care outcomes across multiple contexts. With the growing burden of chronic diseases, healthcare providers, health policymakers, and other healthcare system stakeholders are re-examining their strategies and opportunities for more effective prevention and clinical interventions. The study aimed to find the best practice guidelines and policies influencing effective intervention and making it possible to personalize prevention strategies. Apart from clinical treatment, it is essential to increase the effectiveness of non-clinical interventions that could empower chronic patients to increase their involvement in therapy. The review focuses on the best practice guidelines and policies in non-medical interventions and the barriers to and facilitators of their implementation into everyday practice. A systematic review of practice guidelines and policies was conducted to answer the research question. The authors screened databases and included 47 full-text recent studies in the qualitative synthesis.

## 1. Introduction

Chronic diseases, also known as non-communicable diseases (NCDs), result from a combination of genetic, physiological, environmental, and behavioural factors, and they are most often long-lasting. The main types of chronic diseases include cardiovascular diseases, cancers, chronic respiratory diseases, and diabetes [1]. NCDs are the world’s leading causes of death and disability, with cardiovascular diseases (CVD) accounting for half of the deaths caused by NCDs. A meaningful way to control chronic diseases is to reduce the risk factors associated with these diseases. NCDs kill 41 million people yearly, corresponding to 71% of all deaths globally. About 422 million people worldwide have diabetes, most of whom live in low-and middle-income countries, and 1,6 million deaths are directly attributed to diabetes each year. The number of cases and the prevalence of diabetes have been steadily increasing over the past few decades. This number expected to rise to 578 million by 2030 [1]. Cardiovascular diseases (CVDs) are the number 1 cause of death globally, taking an estimated 17.9 million lives each year. Four out of 5 CVD deaths are due to heart attacks and strokes, and one-third of these deaths occur prematurely in people under 70.

To support countries in their national efforts, the World Health Organization developed a ‘Global action plan for the prevention and control of NCDs 2013–2020’, which includes nine global targets that have the greatest impact on global NCD mortality. These targets address the prevention and management of NCDs and include (1) A 25% relative reduction in risk of premature mortality from cardiovascular diseases, cancer, diabetes, or chronic respiratory diseases. (2) At least a 10% relative reduction in the harmful use of alcohol, as appropriate, within the national context. (3) A 10% relative reduction in the prevalence of insufficient physical activity. (4) A 30% relative reduction in mean population intake of salt/sodium. (5) A 30% relative reduction in the prevalence of current tobacco use in persons aged 15+ years. (6) According to national circumstances, a 25% relative reduction in the prevalence of raised blood pressure or contain the prevalence of raised blood pressure. (7) Halt the rise in diabetes and obesity. (8) At least 50% of eligible people receive drug therapy and counseling (including glycaemic control) to prevent heart attacks and strokes. (9) An 80% availability of affordable basic technologies and essential medicines, including generics, is required to treat major non-communicable diseases in public and private facilities [2]. Hypertension guidelines are necessary for proper and adequate prevention, early detection, evaluation, treatment, and control of hypertension [3]. 

WHO (2016), in the Action Plan for the Prevention and Control of Non-communicable Diseases in the WHO European Region, prioritises population-level interventions, i.e.:

1. Promoting healthy consumption via fiscal and marketing policies
2. Product reformulation and improvement: salt, fats, and sugars
3. Salt reduction
4. Promoting active living and mobility
5. Promoting clean air by reducing outdoor and indoor air pollution

The other priority is individual-level interventions, i.e.:

1. Cardio-metabolic risk assessment and management
2. Early detection and effective treatment of significant NCDs
3. Vaccination and relevant communicable disease control

In September 2021, the WHO published the Discussion Paper on developing an implementation roadmap 2023–2030 for the WHO Global Action Plan for the Prevention and Control of NCDs 2023–2030. One of the priorities on the roadmap should be understanding the drivers and trajectories of NCD burden across countries and epidemiological regions [4]. Countries should systematically examine their progress in introducing evidence-based national guidelines, protocols, and standards for managing NCDs, including policies for NCD research and inclusion and consideration for vulnerable groups. 

WHO experts in the same document pay attention that not all barriers identified on a global scale are relevant in all settings, and countries should seek to prioritize and address those specific to their local context [4]. In line with the WHO recommendations for the priorities on the 2023–2030 roadmap [4], the focus should be on understanding the drivers and trajectories of NCD burden across countries and epidemiological regions. 

Compared to previous years, an impressive increase in interest in the subject of personalized medicine in the context of effective intervention plans and implementation has been noticed in the last decade. However, the authors identified a lack of systematic research in the current chronic disease prevention and control literature. Poitras et al. [5] claim that elements of interventions can be grouped into three main types and clustered into seven categories of interventions: (1) Supporting decision process and evidence-based practice; (2) Providing patient-centered approaches; (3) Supporting patient self-management; (4) Providing case/care management; (5) Enhancing interdisciplinary team approach; (6) Developing training for healthcare providers, and (7) Integrating information technology. Their scoping review provides evidence for the adaptation of patient-centered interventions for patients with multimorbidity. 

Baugh Littlejohns and Wilson [6] reported seven attributes of effective systems for chronic disease prevention: collaborative capacity, health equity paradigm, leadership and governance, resources, implementation of desired actions, information, and complex systems paradigm.

In a systematic review with narrative synthesis ‘Implications of interprofessional primary care team characteristics for health services and patient health outcomes, Wranik et al. [7] highlight the role of interprofessional primary care teams as an alternative to single profession physician practices in primary care with a focus on preventive care and chronic disease management. They argue that researchers should focus on quantitative causal inferences about linkages between team characteristics and patient health.

Haregu et al. [8] presented a very interesting focus in a scoping review of non-communicable disease research capacity-strengthening initiatives in low and middle-income countries. They argue that most initiatives focus on building individual capacity, and only a few focus explicitly on institutional-level capacity strengthening. Though many of the initiatives appear to have had promising short-term outcomes, there is a lack of evidence of their long-term impact and sustainability.

A review by Reynolds et al. [9] demonstrated the benefits of implementing interventions based on chronic care model elements in primary care. Their findings provide further evidence to support the view that self-management education should be an integral part of high-quality primary care [10]. 

In their review of 28 hypertension guidelines (written or translated into English), Owolabi et al. [11] claim that every intervention must meet essential criteria, including validity, reliability/reproducibility, clinical applicability, clinical flexibility, socioeconomic and ethical-legal contextualisation, clarity, multidisciplinary process, scheduled review, and rigorous dissemination plan [12]. Unfortunately, none of the available guidelines they reviewed meets all these criteria. According to the authors, this could explain why hypertension is still difficult to control in many regions of the world, as possible valuable channels for disseminating and implementing guidelines are not harnessed. They also implicate those efforts are needed to develop hypertension guideline(s) for Low and Middle-Income Countries (LMIC). The expected guideline(s) should be broad-based, flexible, adaptable, socio-culturally acceptable, and economically attainable for better health-related outcomes in patients with hypertension [11]. 

This review partially contributes to all the reviews mentioned above. It adds to the base through deep analysis of the barriers and facilitators that influence the implementation of best practices and policies at particular intervention delivery levels in the micro-, meso-, and macro-environment of the chronic disease. 

Well-developed guidelines for primary care management are often inadequately operationalised. While experienced healthcare providers can often adapt the guidelines to their contexts, how they do this and how they learn what works is not understood or recorded. The authors focus is looking for the barriers and facilitators that could accelerate effective intervention implementation.

This review’s primary objective is to summarise the existing literature related to barriers and facilitators in the implementation of prevention strategies for chronic patients by using reproducible and explicit approaches to identification, appraisal, and synthesis of included sources. The authors seek to understand the barriers and facilitators that support or hinder the intervention implementation process of best practice guidelines and policies. The analysis is made for the micro-, meso-, and macro-intervention delivery levels. The full texts were screened to identify the barriers and facilitators, and then the quality assessment of the content was made. The authors identified the need to group the outcome into the levels of delivery to provide the best recommendations for each stakeholder of the healthcare process.

## 2. Materials and Methods

The systematic review was done according to PRISMA recommendations and rules [13]. The procedure of doing the systematic review included preparing a detailed Review plan approved by two independently working researchers and searching two databases using the agreed keywords. The review protocol described the review’s rationale, hypothesis, and planned methods. It had been prepared before a review started and used as a guide to carry out the review. In the next step, the co-authors performed the initial search of the literature (scoping search) independently. 

The search process covered the following keywords: chronic disease, prevention, practice, and policy. Two publicly available/free resources were used: PubMed and Google Scholar. The authors of the review focused on the most recent sources covering the years 2016 to 2022. The search in the bases was limited to the sources in English. Also, manual searching was carried out. Manually searching was focused on searching for the synonyms to the defined keywords/MesH phrases. The authors also searched for papers using the words: intervention, program, initiative, and non-communicable disease. The manuscript refers only to the published data. 

As a result of the initial search,524 results were obtained from PubMed. Keywords for searching that were used: chronic disease + prevention + practice+ policy. The results included 24 clinical trials, 23 randomised controlled clinical trials, 13 meta-analyses, 75 reviews, 30 systematic reviews, and 3 books and documents. Google Scholar processed 16,900. Keywords used for searching: chronic disease + prevention and control + practice guideline + policy. 

Records identified through database searching n = (1) 524 + (2) 16,900

The screening covered the following:

1. Records screened (1) + (2) by titles: 17,424
2. Records screened by title for a detailed reading of abstracts n = 86
3. Abstracts excluded with reasons: n = 37

Eligibility

1. Full texts were assessed for eligibility after the detailed reading of abstracts and after deleting duplicates n = 49
2. Full texts excluded with reasons: the trial is not completed n = 2

Records included

1. Full-text studies included in qualitative synthesis n = 47

**Figure 1:**
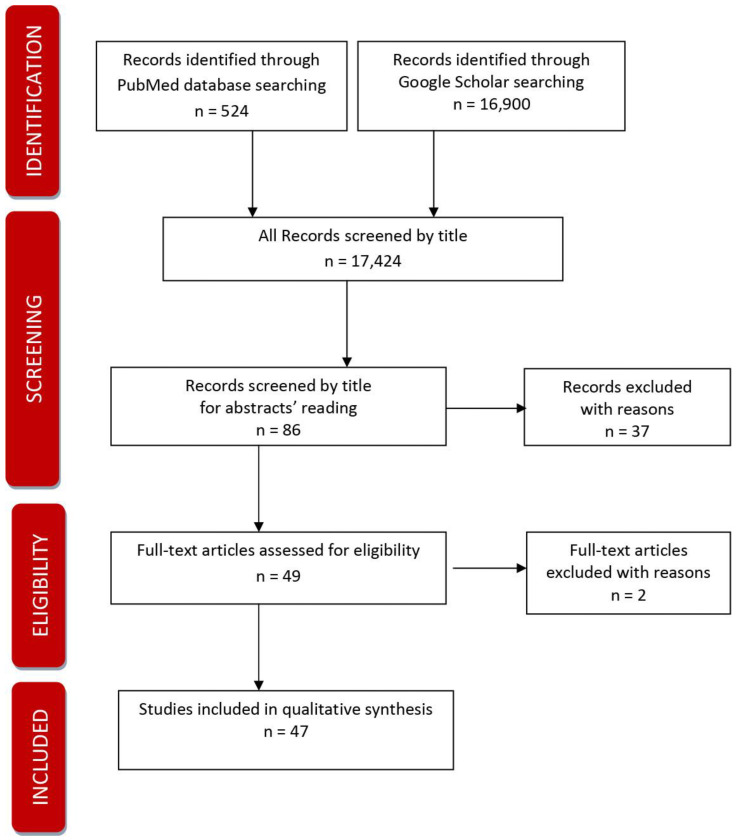
PRISMA diagram.

The Figure 1 below presents the applied PRISMA process.

Once the titles were identified in the bases searching and manual searching, the authors decided on the eligible paper inclusion in the three-step process:

1. screening for abstract level
2. screening for full texts level
3. manual searching level

The title and abstract and, after that, the full text of the articles were screened by the two authors. The eligible article was supposed to:

1. focus on prevention and control strategies/interventions
2. address a chronic disease
3. focus on practice guidelines or policies

The selection of papers was based on the PRISMA statement. Critical (quality) appraisal was made on 47 carefully selected papers. The review included broad global literature, the studies cover guidelines, recommendations, and strategies developed by North and South American, European, Australian, African, and Asian researchers. Most papers relate to cross-country studies. 

The following exclusion criteria were used to select the most appropriate papers:

1. the article relates to disease treatment, not prevention and control
2. the article does not relate to non-communicable/chronic disease
3. the trial is not completed

In case the authors have different opinions about the inclusion of some studies, the consensus was achieved in the discussion process. There were two rounds of negotiations—at “screening for abstract level” and “screening full text according to eligibility criteria level”. The number of studies selected for the deep analysis was compromised, resulting in this negotiation and critical appraisal.

Once the inclusion and exclusion criteria were applied, the selected studies were assessed in detail according to their quality, and the content was analysed and interpreted systematically.

The Cochrane acronym PICO (for population, intervention, comparison, outcomes) was helpful in ensuring that the decision on all critical components was made before starting the review. 

The primary target group of the study is the growing population of chronic patients and patients at risk of chronic disease. 

The second target group is other stakeholders who are involved in the patient’s journey toward better well-being:

1. Professional healthcare providers: physicians, family and community nurses, community and social workers, etc.
2. The patients’ caregivers are formal and informal—family members, neighbours, etc.
3. Healthcare organisations: hospitals, clinics, and nursing homes that provide infrastructure and other complementary resources to support the work and development of care teams,
4. Policymakers and stakeholders involved in healthcare: patient associations, representatives of NGOs, representatives of local governments involved in shaping regional health policies, and legal representatives of healthcare care providers.

Barriers and facilitators to implementation may arise at multiple levels of healthcare delivery: micro-, meso-, and macro-level [14]. Following this narrative, the authors systematized the research in the following levels of intervention delivery:

I. Micro-level would refer to the individual stakeholders of the healthcare system: i.e., already diagnosed patient or at-risk patient, the care partner, the healthcare provider;
II. Meso- level would refer to the organisational level: i.e., hospital, clinic, and nursing home that provide infrastructure and other complementary resources to support the work and development of care teams and micro-systems;
III. Macro-level would refer to the market/policymakers level: i.e., regulatory, financial, and payment regimes and entities that affect the structure and performance of healthcare organisations.

## 3. Results

Based on the quality analysis of the selected texts, the authors categorized the studies into the following 14 categories described in Table 1. below. The critical appraisal was conducted, and the authors made the decision to classify the 47 studies to highlight the current literature focus and trends in the best practice guidelines and policies.

Barriers and facilitators to implementation that may arise at multiple levels of healthcare delivery: micro-, meso-, and macro-level are presented in Table 2 below. 

The authors screened databases and included 47 full-text recent studies in the deep qualitative synthesis.

## 4. Discussion

### 4.1. Chronic Disease Prevention and Management

The prevention and management of chronic conditions are critical in healthcare globally. Evidence-based recommendations for the screening and management of chronic conditions have been developed, but the patient outcome and reach evaluations have not always been positive. This is frequently due to a lack of proper translation of guidelines and patients’ non-compliance with advice and adherence to recommendations. General practice is overwhelmed with clinical guidelines, and implementing all of them may result in significant polypharmacy, despite their utility [51,52].

The study confirms that effective healthcare management strategies should engage the health professionals, the patient himself and his local environment in decision-making and guarantee the employment of guidelines in any organisational context. 

Prevention and treatment of chronic diseases is a global challenge in public health. Engaging patients and caregivers is a critical factor for effective interventions. Undertaking actions on each level of the intervention delivery—either micro-, meso-, or macro—improves treatment schemes; however, it is worth mentioning that too rapid or too complicated innovations may be the roadblock to improvement. Preventing chronic diseases requires complex interventions involving multi-component and multi-level efforts tailored to the context in which they are delivered [53]. 

### 4.2. Personalisation Strategies

There are well-established associations between behaviour and chronic diseases, which justify government efforts to reduce behavioural risk factors. However, the question of how population behaviour patterns might be shifted most effectively remains one of the most significant research and policy uncertainties [54]. What is essential in the implementation of priority interventions to make them effective is one of the leading research questions of the last decades.

Some researchers have criticised the “cookbook” approach that guidelines may promote [55]. In such opinions, evidence-based medicine may undervalue the tacit knowledge of healthcare providers, which comes from their experience and relates to the context in which they work. Any guidelines, in addition to being based on clinical evidence, need to be flexible, adaptable, socially and culturally acceptable, and economically attainable for better health-related outcomes in patients.

Research evidence does not automatically diffuse into clinical practice but requires active translation that starts with clinicians’ awareness of the science and ends with patient adherence to the recommended care. Scott &Glasziou [50] claim that cognitive, motivational, and sociological factors on the part of health professionals are critical in this process. Many studies highlight awareness’s role in achieving the best efficacy of the intervention in terms of the patient’s understanding, caregivers’ awareness and healthcare professionals’ awareness. Guidelines, in addition to being based on clinical evidence, need to be broad-based, flexible, adaptable, socially and culturally acceptable, and economically attainable for better patient health-related outcomes. As exemplified by the National Institute for Clinical Excellence guidelines, patients’ participation should be incorporated to enhance adherence to these recommendations. Indeed, the active involvement of all stakeholders in the design of guidelines will likely improve implementation and effectiveness (11).

Regular monitoring and evaluation, with defined and shared outcomes and indicators, are essential for further programme implementation using quantitative and qualitative methods [30].

### 4.3. Importance of Qualitative Studies

The qualitative analysis of the eligible sources chosen for the review and presented in Table 2. points to the balanced importance of healthcare delivery levels. There is a need to increase the capacity of all systems, on micro-, meso-, and macro-level collectively, to bring the most effective practical results. The most critical advance in chronic patient prevention strategies would be to put together the activities at all levels of delivery in the form of thoughtful, integrated pathways that would be tailored to the context and able to be scaled up. Stakeholders of the prevention process should be involved in the process from the planning phase through the implementation process.

There is no “one size fits all” solution, particularly in the non-standard situations that produce the health inequities on which we will focus. It is based on the premise that the way forward is to create tools, guidelines, and materials for training methods and skills that will enable healthcare practitioners to design their multi-component interventions that will be person and context-based.

The theoretical model of adaptive implementation [14] describes external factors (e.g., characteristics of the intervention, operational preconditions, personal and financial resources) that can affect the implementation of interventions during various phases (preparation, execution, and continuation). It differentiates between influencing factors on different levels in each of these phases: micro-level (user/primary process), meso-level (inter-organisational/social context), and macro-level (healthcare system, legislation, policy). 

Undoubtedly, an evidence-based approach to prevention can significantly minimise chronic disease burden. There is a strong need for evidence derived from complex intervention evaluation methodologies in diverse health and social care contexts [56]. 

### 4.4. Barriers and Facilitator Identification

It is necessary to identify the barriers to and facilitators of the implementation process to increase its effectiveness. The global target is to identify high-impact interventions and identify barriers to their implementation and opportunities for acceleration. 

Barriers to implementation may arise on multiple levels of healthcare delivery: micro-, meso-, and macro-level. Micro-level refers to the patient level (already diagnosed patient and an at-risk patient), the care partner level, and the healthcare provider level. Meso- level would refer to the organisational level, while macro-level to the market/policy level. 

Multi-sectoral partnerships (MSPs) are frequently cited as a means by which governments can improve population health while leveraging the resources and expertise of private and non-profit sectors [57]. The social, psychological, and economic situations of regions or countries should be considered while deeper analysing healthcare systems. Reducing the burden of chronic diseases is a global challenge requiring diverse collaborations and the diffusion and adoption of effective interventions in multiple settings. The past decade has seen various innovative community-driven and clinically driven primary and secondary prevention strategies designed to prevent and reduce the burden of chronic conditions worldwide [58]. Current literature points out more often the significance of health literacy that influence the process of chronic disease prevention and treatment.

### 4.5. Limitations of the Review

The current study review has some limitations. Two databases were searched on the base of slightly different scopes. The Google Scholar search was narrowed to hypertension and diabetes to obtain the most precise result. Narrowing the PubMed search by analogy resulted in significant limitations in available sources. The authors decided to adjust the scope of the search to the potential of the databases. Second, the article was limited to the open access published articles (free full texts). It was probable that this review could have omitted some eligible studies. To overcome this limitation, manual searching was performed.

### 4.6. Directions for Future Research

Further research on the effectiveness of innovation implementation/health technology assessment will be required– innovation either improves or harms the health system (exnovations), including patient care experience, quality of care, and cost of care [59]. More advanced research will also be recommended on preventing multiple chronic conditions on the fiscal level (to avoid catastrophic health spending), inter-sectoral level, and more robust practice evidence level.

Public health interventions that were found effective in selected need to be scaled up and implemented more widely to achieve population-wide improvement. The pathways through which interventions are scaled up are not sufficiently characterised [42]. It would be recommended to perform a deeper analysis of scaling-up procedures and barriers to and facilitators of implementing scaling-up pathways.

For the best output, there should be a focus on patient empowerment via patients’ associations and cooperation with policymakers and healthcare service payers, involving them from design to evaluation and final scaling up of the intervention. The efficacy of implementation should be monitored continuously to look for the strategic barriers and facilitators of the process implementation.

## 5. Conclusions

In the existing literature, there is a considerable record of the best practices and consequences of lack of adherence to the recommendation. Current health policy focuses on chronic conditions, which cannot be cured but managed through medication and/or other therapy or further complications prevented by modifiable lifestyle factors. As chronic diseases are associated with an increasing disease burden globally, it is crucial to reflect on the efficacy of the existing policies and practices. There is little evidence of barriers and facilitators of implementing particular practice guidelines into everyday practice. The gaps the authors filled by conducting this systematic review are a qualitative analysis of the barriers and facilitators that affect the translation of best recommendations into everyday medical practice.

The results of this review provide a framework to strengthen chronic disease prevention, especially in terms of evidence-based and practice-based recommendations for health systems. Most literature sources postulate that collaboration among professionals and their organisations increases the health system’s capacity, and the involvement of patients and policymakers in developing guidelines may affect the implementation. The number of reviews also underlines the importance of technology in the intervention implementation process. This review is also in line with the existing literature, which highlights the role of increasing awareness of healthy lifestyle recommendations, especially among populations where the health literacy level is not sufficient.

Complementary to the existing literature, we sought a deeper understanding of the barriers and facilitators that help to empower patients through their behaviour change process. Health behaviour change is not just a personal issue; instead, it is grounded in a system of psychological, social, and environmental factors, the full context of the patient. Behaviour change cannot be considered a purely personal process but a system process, where professionals, together with the person, address internal and external factors and their interactions. Contextual, relationship and social factors will be integrated into the intervention delivery as possible influential barriers or facilitators.

## Figures and Tables

**Table 1:** Category of the priority focus in the selected studies.

	Category	Description
1	Life style	The study highlights the role of lifestyle and modifiable risk factors, i.e., physical activity, dietary recommendations, etc. The study discusses the behaviour change process
2	Social factors	The study highlights the role of social factors and the whole patient’s environment in the process of his recovery
3	Economic factors	The study highlights the role of economic factors in the effectiveness of health interventions
4	Resources	The study highlights the importance of the resources’ availability for the healthcare system in terms of human and technical resources
5	Awareness	The study highlights the role of various kinds of awareness in the process of achieving the best efficacy of the intervention
6	Health literacy	The study highlights the significance of health literacy in the process of achieving the best efficacy of the intervention
7	Patient engagement	The study highlights the role of patient and/or at-risk patient’s engagement in the process of prevention and control, and study addresses the role of patient’s adherence to recommendations
8	Healthcare provider	The study reflects on the professional healthcare provider’s role in the chronic patient journey toward better well-being
9	Caregiver	The study reflects on the non-professional caregiver’s role in the chronic patient journey toward better well-being
10	Policymakers	The study reflects on the role of policymakers in the patient’s journey toward better well-being
11	Networking	The study highlights the role of networking, interdisciplinary cooperation, and communication between all stakeholders
12	Social campaigning	The study highlights the role of social campaigning for chronic disease prevention and control
13	Technology	The study highlights the role of technology development in the healthcare system efficacy
14	Local context	The study highlights the significance of contextualisation, especially in terms of vulnerable populations

**Table 2:** Barriers to and facilitators of intervention implementation concerning the level of intervention delivery.

Level of Intervention Delivery	Barrier		Facilitator	
Micro-level	6	Lack of health literacy in society is recognised as a determinant of health [15,16]	12	Targeted strategy to increase awareness, treatment, and control in individuals [17] healthcare professionals’ awareness of challenges, patients gaining greater awareness [15,16]
	8	Unclear professional boundaries, low compensation level, insufficient knowledge and capabilities [18]	11	Optimising the prevention, recognition, and care of hypertension requires a paradigm shift to team-based care [17]
	8	Disregarding patient’s preferences for different health outcomes [19]	7	Meaningful patient involvement [20] patient self-management, patient-centered approach [5,9]
	5	Community perception—lack of awareness of diabetes risk factors [21]	6	Patient’s Health Information Seeking Behaviours—increase empowerment/focus on control, and satisfaction [22]
	8	Competences, motivation, and workload professionals [23]	1	Reduction of unhealthy behaviours and risk factors such as tobacco use and obesity [24,25]
	1	Unhealthy behaviours and risk factors such as tobacco use and obesity [24,25]	11	Engaging patients and stakeholders around multiple chronic conditions could improve the relevance of clinical practice guidelines [26], care management [5] interdisciplinary team approach [5]
	8	Not sufficient training for healthcare providers [5]	1	Physical Activity and Sedentary Behaviour, Activities of daily living, and health outcomes [22]
			9	Support from the caregiver, awareness of the caregiver [27]
Meso- level	8	Insufficient provisions of preventive services within primary healthcare and inappropriate referrals to ambulatory care [28]	14	Sustainability and scalability of pilot actions [20]
	4	Experiencing uncertainty among staff when implementing new programmes—multi-sectoral partnerships for chronic disease prevention [1]	13	Information technology [5]
	2	Unsupportive organisational and institutional environment [18]	4	Toolbox for the design and implementation of selective prevention initiatives [29]
	4	Unclear description of care pathways, addressing specific groups and the areas of health promotion [30]	5	Identification of a significant disease cluster [31]
	4	Obstacles to inpatient hospital access [32]	11	Applying managed care models [33] Developing and structuring cross-sector relationships [34] Well-established coordination and collaboration, collaborations across the boundaries of organisations [23,35]
	11	Lack of proper communication and information [23]	11	Increasing staff involvement at the social context level may minimise barriers due to a lack of communication and cooperation [36]
	10	Hospital specialists and clinic GPs disagree on Clinical Practice Guidelines [32]	11	A vertically integrated service model could optimise the care and shift the care from hospital to primary care [37]
	11	Not engaging the community in the process of developing and introducing any new programmes [38]	6	Inter-professional practice and education to address gaps in care [27]
	4	Limited resources including funding and the number of staff [21,23]	11	Good teamwork: shared space, common vision and goal, clear definitions of roles and leadership [7]
Macro-level	6	System-level leadership to ensure that curricula for healthcare workers’ training contain information on the importance of health literacy in their clinical practice, health system administrators provide signage and educational materials that are at appropriate literacy levels and representative of the languages and cultures of patients [15]	10	Regular exercising and reducing sedentary behaviours through policies to inform national health policies and strengthen surveillance systems that track progress towards national and global targets [39]
	10	Prevention has not collated the tacit knowledge of diverse actors in a structured way—lack of concept mapping [40]	14	The administrative evidence-based practice facilitates the role of public health departments in implementing the most effective programmes and policies [41]
	5	Understanding pathways for scaling-up public health interventions [42]	11	Collective sharing of challenges and opportunities and learning across countries [43]
	5	Most initiatives focus on individual-level capacity and not system-level capacity [8]	11	Co-creation [44]
	4	Fragmentation and misalignment of healthcare systems [35] Lack of framework to help strengthen systems [6]	12	Population-level evaluation and systematic media follow-up [30]
	4	Popularity and funding availability as opposed to effectiveness [45]	10	Political support, alignment with current healthcare trends, ongoing technical improvements, and capacity building [46]
	10	Conventional care prioritises maternal and child health, neglecting adult chronic diseases [30]	14	New models should be built on a bottom-up and dynamic approach based on local needs, resources, and initiatives [29]
	4	Lack of human resources to respond to a growing demand for healthcare services for adult patients [32]	11	All national and local partners and stakeholders should be involved from the beginning of the planning phase, and partnerships should be kept active throughout the process [30]
	4	Lack of necessary equipment to control chronic diseases such as diabetes and hypertension [32]	11	Highlighting the importance of administrative evidence-based practice to the public health leadership level may enhance practice [41,47]
	4	Shortages of free medication to treat chronic patients [32]	10	
	10	Lack of functional accessibility and gender bias [32]		
	10	Improper implementation—ending effective programmes prematurely or continuing ineffective ones [48]		Government leadership: government-led, leadership-oriented implementation is the core for the prevention and control of chronic diseases [49]
	10	Pre-emption [50]		

## Data Availability

Not applicable.
